# Application of tele-neuropsychology and tele-mental health before and during COVID-19 era: a bibliometric analysis

**DOI:** 10.1097/MS9.0000000000001822

**Published:** 2024-02-19

**Authors:** Abraish Ali, Rameen Zafar, Kanwal Ashok Kumar, Kainat Shariq, Vanita Motiani, Sufyan Ibrahim, Hadi Farhat

**Affiliations:** a Department of Medicine, Dow Medical College, Karachi, Pakistan; b Department of Internal Medicine, Western Michigan University Homer Stryker School of Medicine, Kalamazoo, MI; c Department of Neurosurgery, Mayo Clinic, Rochester, MN; d Department of Medicine, Lebanese University, Beirut, Lebanon

**Keywords:** COVID-19, neuropsychiatry, SARS-COV-2, tele-mental health, tele-neuropsychology

## Abstract

**Background:**

Telehealth use was previously limited by strict regulations and financial constraints. However, the pandemic prompted some countries to ease their telehealth laws. Thus, we conducted a bibliometric analysis and network visualization to gauge changes in tele-neuropsychology (Tele-NP) and tele-mental Health (Tele-MH) trends with the onset of the COVID-19 pandemic.

**Materials and methods:**

The authors conducted a literature search on SCOPUS and included relevant articles pertaining to Tele-NP and Tele-MH, which were published before the COVID-19 pandemic (2017–2019) and during the COVID-19 pandemic (2020–2022). The authors presented publication patterns on psychiatric disorders, mode of administration, journals, active countries, authors, affiliations, funding sponsors, keywords, publication, and citation output.

**Results:**

Three hundred forty-one articles were included in our study with 80 articles before the pandemic and 261 during the pandemic. Our analysis revealed the greatest increase in publications and citations output was from the year 2020 to 2021. A greater number of journals published tele-NP and tele-MH-related research, with higher frequency, during the COVID-19 pandemic with Telemedicine and E-health leading the way. WHO regional analysis revealed that the Region of the Americas (AMRO) was the leading contributor in terms of publications. Harvard Medical Center remained the number one contributor both before and during the COVID-19- pandemic. Publications by funding sponsors, particularly by those that were US-based, increased twice as much during the pandemic.

**Conclusion:**

The increase in research output following the COVID-19 outbreak reflects the growing interest and relevance of telemedicine for the delivery of mental health services.

## Introduction

Highlights

- No bibliometric analysis on trends in tele-neuropsychology and tele-mental health research output after the pandemic
- The Region of the Americas was the leading contributor for publications.
- US-based funding sponsors published twice as much during the pandemic.
- Increased research on anxiety, depression, and trauma-related stress during the pandemic.
- To realize the promise and perils of telehealth going into the future, further studies documenting patient expectation and satisfaction for telehealth are needed.

Tele-neuropsychology (Tele-NP) and tele-mental health (Tele-MH) became unquestionably essential for the global management of COVID-19. Prior to the COVID-19 pandemic, several countries imposed strict legal regulatory measures to ensure accountability, ethical medical practice, and patient data privacy^1^. However, with the onset of the pandemic, many regulations governing the use of telehealth were relaxed to facilitate its broader adoption. In the United States, Medicare adjusted its limitations on geographical and platform restrictions, permitting reimbursement for telephone-only visits, whereas previously only audio-visual methods were covered. Additionally, prior to the pandemic, telehealth services were confined to approved platforms; however, with the onset of COVID-19, restrictions were eased to encompass popular options like Zoom, Skype, and FaceTime^2^. This enhanced accessibility significantly increased the likelihood of enhanced medical care, diagnosis, and reduced hospitalization duration for non-serious conditions^3,4^. Patients could receive higher- level virtual medical support before potential hospitalization, potentially bypassing the Emergency Department and going directly to a hospital bed^5^. The discrepancy in accessibility was apparent in Health Center Program Data from 2019 where 43% of health centres were able to provide telemedicine services compared with 95% utilizing telehealth during the COVID-19 pandemic^6^.

The US government invested $2.3 billion in mRNA COVID-19 vaccine research from 2020 to March 2022, including $1.7 billion for Moderna’s clinical trials (BARDA), $490 million for NIH-funded trials, and $108 million for manufacturing and science investments^7^. Recognizing the accelerated adoption of tele- NP and tele-MH during the COVID-19 pandemic, we conducted a comprehensive bibliometric analysis and network visualization spanning the periods before (2017–2019) and during the pandemic (2020–2022). This investigation was prompted by the urgent need to understand emerging trends, challenges, and opportunities associated with the rapid integration of tele-NP and tele-MH technologies^8^. By systematically examining and visualizing these trends: journals, authorship, affiliations, countries, funding sources, article types, keywords, subject area, citations, and number of publications related to tele-NP or tele-MH before the pandemic (2017–2019) and during the pandemic (2020–2022), our research sought to contribute valuable insights to guide future studies, policy decisions, and the integration of tele-NP and tele-MH into healthcare systems during this transformative period in global health, beyond the pandemic. To the best of our knowledge, there is no bibliometric analysis discussing the impact of the COVID-19 pandemic on tele-NP and tele-MH services, necessitating further research to bridge the gap.

## Methods

### Literature search and strategy

Scopus was the database used to retrieve the relevant documents for this current bibliometric analysis. We searched from January 2017 till October 2022 for the relevant documents without any language and regional restrictions. We used the following search terms “Teleneuropsychology”, and/or “Telemental health”. The literature search was categorized into two different time periods 1) before the COVID-19 pandemic (2017–2019) and 2) during the COVID-19 pandemic (2020–2022).

### Inclusion and exclusion criteria

We included all relevant articles that described the application of tele-NP and tele-MH before (2017–2019) and during (2020–2022) the pandemic. Irrelevant articles unrelated to tele-NP or tele-MH, guidelines, erratum, recommendations, and protocols were excluded from our analysis.

### Data extraction

Two independent reviewers (A.A. and R.Z.) manually screened and assessed all the relevant articles first on the basis of title and abstract, thenceforth full-text review of those articles was done. The data exported from the Scopus contained author names, source titles, year of publication, affiliations, funding sponsors, number of citations, study design, country, and access (open or close). A discussion was carried out on resolving any further discrepancies with K.A.K. and K.S. The concordance rate between the reviewers was 95%.

### Data analysis

**Figure 1:**
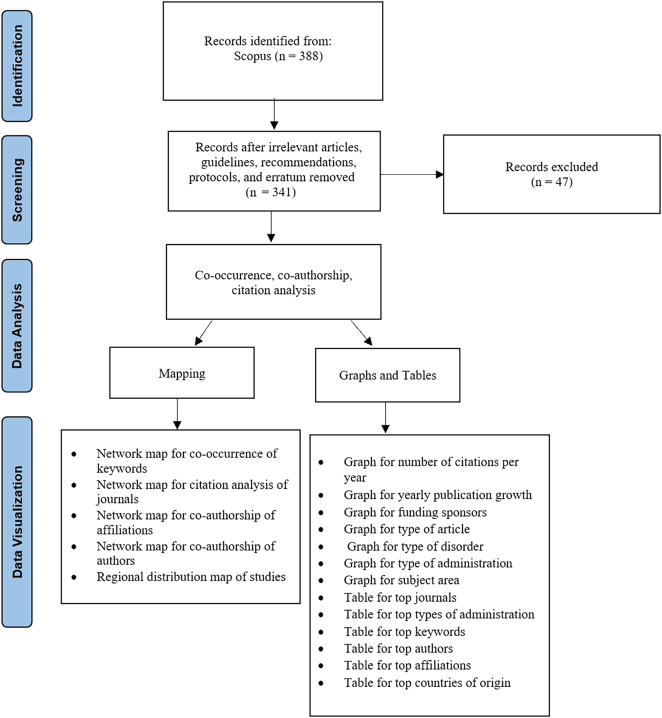
Flow diagram of search process.

In this study, we presented publication patterns on journals, active countries, authors, affiliations, funding sponsors, keywords, publication, and citation output, document type, and subject area (Fig. 1). All trends were ranked on the basis of the frequency of documents. If two categories had the same frequency, the one with the greater total link strength (TLS) took precedence.

The retrieved data was also exported to VOSviewer (version 1.6.18) software^9^ to create a network visualization map of keywords, journals, authors, and affiliations for the two different timelines. Different types of analyses were used for the units of analysis on the VOSviewer program. Keywords were analyzed based on co-occurrence, journals on citation and affiliations, authors and countries were analyzed based on co-authorship. On the network maps, the size of the node indicates the frequency of occurrence, and the distance between two nodes indicates their correlation Fig. 1.

Additional analysis was conducted for the types of psychiatric disorders and administration implemented through tele-MH. The following study types were included in the analysis: randomized controlled trials (RCTs), retrospective and prospective studies, reports, surveys, and pilot studies. We categorized neuropsychology disorders using the diagnostic and statistical manual of mental disorders (DSM-5) criteria^10,11^. If an article described multiple DSM-5 disorders of type of tele-NP or tele-MH administration, these were counted separately.

For geographical distribution of documents, the WHO regional classification was used: the region of the Americas (AMRO), the European region (EURO), the Western Pacific Region (WPRO), the Eastern Mediterranean region (EMRO), the South-Eastern Asia region (SEARO), and African region (AFRO). A geographical map was created on Microsoft Excel for the distribution of countries.

## Results

### Literature search

The literature search of the Scopus database yielded 388 results initially; all the titles and abstracts were screened. As a result, we excluded 47 articles and included 341 in our study, comprising of 80 before the pandemic and 261 during the pandemic (Fig. 1).

### Tele-NP and tele-MH disorder categories classified by DSM-V criteria

**Figure 2:**
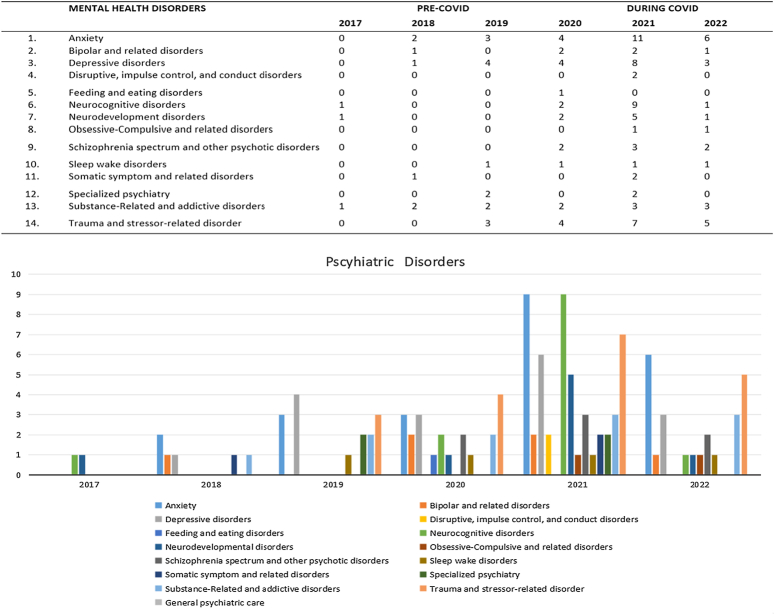
Tele-neuropsychology and tele-mental health disorders classified by diagnostic and statistical manual of mental disorders (DSM-5) criteria before (2017–2019) and during the COVID-19 pandemic (2020–2022).

A total of 120 studies explored tele-NP or tele-MH usage across 14 psychiatric disorder categories. The peak year for identifying psychiatric disorders was 2021. The most frequently cited DSM-V disorders during the pandemic were trauma stressor-related disorders (*n*=16, 16.49%), anxiety (*n*=23, 23.71%), and depression (*n*=17, 17.52%). Moreover, schizophrenia (*n*=7,100%), neurocognitive (*n*=12, 92.3%), and substance-related addictive (*n*=8, 72.72%) increased substantially during the pandemic as well. Notably, the number of feeding/eating and sleep-wake disorders did not drastically change during the pandemic (Fig. 2).

### Type of tele-NP and tele-MH administration

Among 341 documents, 138 reported different types of administration. The use of various administration types increased between 2020 and 2022, with a significant surge in 2021 (eFigure S5, Supplemental Digital Content 1, http://links.lww.com/MS9/A383). Before COVID-19, tele-NP and tele-MH were mainly used for diagnostic assessment (*n*=5; 16.12%), education/training (*n*=4; 12.90%), and counselling (*n*=4; 12.90%). During the pandemic, tele-MH was frequently used for cognitive assessment (*n*=22; 20.56%) and obtaining feedback/surveys about users’ and practitioners’ satisfaction (*n*=20; 18.69%).

### Citation analysis of Journals

Before the COVID-19 pandemic, Current Psychiatry Reports (*n*=6, 7.5%) and Telemedicine And E-Health (5 articles, 6.3%), were the top contributing journals (Table 1). Meanwhile, Telemedicine And E-Health (*n*=20, 7.7%) and Archives Of Clinical Neuropsychology (*n*=13, 5%) were the top journals during the COVID-19 pandemic (Table 1). The average mean Impact Factor (IF) of journals was slightly higher during COVID-19 (IF= 4.6) compared to before COVID-19 (IF= 4.4). Before the COVID-19 pandemic, the citation analysis showed that Health Affairs and the Journal of Family Psychology (total strength links= 3) had the strongest connections [eFigure S6 (A), Supplemental Digital Content 1, http://links.lww.com/MS9/A383]. Meanwhile, Clinical Neuropsychologist (total strength links=42) had the strongest connections during the COVID-19 pandemic [eFigure S6 (B), Supplemental Digital Content 1, http://links.lww.com/MS9/A383].

**Table 1:** Top 5 journals that contributed on tele-neuropsychology and tele-mental health research publications before (2017–2019) and during the COVID-19 pandemic (2020–2022).

Rank	Journal	*N* (%)	Impact factor
	Before the COVID-19 pandemic 2017–2019 (*n*=80)		
1.	Current Psychiatry Reports	6 (7.5)	8.1
2.	Telemedicine And E Health	5 (6.3)	5.0
3.	Psychiatric Clinics Of North America	4 (5.0)	2.8
4.	Journal Of The American Psychiatric Nurses Association	3 (3.8)	2.1
4.	Canadian Journal Of Psychiatry	2 (2.5)	5.3
4.	Health Affairs	2 (2.5)	9.0
4.	Issues in Mental Health Nursing	2 (2.5)	1.8
4.	Journal Of Telemedicine And Telecare	2 (2.5)	6.3
4.	Military Medicine	2 (2.5)	1.6
5.	Acta Informatica Medica	1 (1.3)	1.8
5.	Addiction Research And Theory	1 (1.3)	3.0
5.	American Journal Of Geriatric Psychiatry	1 (1.3)	8.0
5.	Archives Of Clinical Neuropsychology	1 (1.3)	3.4
5.	Behavior Therapy	1 (1.3)	4.8
5.	Child And Adolescent Psychiatric Clinics Of North America	1 (1.3)	2.4
5.	Community Mental Health Journal	1 (1.3)	2.5
5.	Duke Law Journal	1 (1.3)	2.7
5.	Evaluation And Program Planning	1 (1.3)	1.9
5.	Families, Systems And Health	1 (1.3)	1.6
5.	Frontiers In Psychiatry	1 (1.3)	5.4
5.	Harvard Review Of Psychiatry	1 (1.3)	3.9
5.	Indian Journal Of Psychological Medicine	1 (1.3)	2.6^a^
5.	International Journal Of Environmental Research And Public Health	1 (1.3)	4.6
5.	International Journal Of Telemedicine And Applications	1 (1.3)	2.1
5.	Internet Interventions	1 (1.3)	5.4
5.	Iranian Journal Of Psychiatry	1 (1.3)	2.6^a^
5.	JAMA Psychiatry	1 (1.3)	25.9
5.	JAMIA Open	1 (1.3)	NA
5.	Jmir Formative Research	1 (1.3)	7.1
5.	Jmir Mental Health	1 (1.3)	6.3
5.	Journal Of Abnormal Child Psychology	1 (1.3)	4.1
5.	Journal Of College Counseling	1 (1.3)	NA
5.	Journal Of Consulting And Clinical Psychology	1 (1.3)	7.2
5.	Journal Of Deaf Studies And Deaf Education	1 (1.3)	2.1
5.	Journal Of Family Psychology	1 (1.3)	3.3
5.	Journal Of Health And Human Services Administration	1 (1.3)	NA
5.	Journal Of Obsessive-Compulsive And Related Disorders	1 (1.3)	2.2
5.	Journal Of Rural Health	1 (1.3)	5.7
5.	Journal Of Social Distress And The Homeless	1 (1.3)	2.6^a^
5.	Journal Of The Canadian Academy Of Child And Adolescent Psychiatry	1 (1.3)	NA
5.	Journal Of Traumatic Stress	1 (1.3)	4.0
5.	Managed Care	1 (1.3)	3.2
5.	Mayo Clinic Proceedings	1 (1.3)	12.2
5.	Neuropsychology Review	1 (1.3)	6.9
5.	Perspectives In Psychiatric Care	1 (1.3)	2.2
5.	Ppmp Psychotherapie Psychosomatik Medizinische Psychologie	1 (1.3)	0.8
5.	Professional Psychology: Research And Practice	1 (1.3)	1.9
5.	Psychiatric Times	1 (1.3)	NA
5.	Psychological Services	1 (1.3)	3.1
5.	Psychotherapy Research	1 (1.3)	4.1
5.	Temple Law Review	1 (1.3)	NA
5.	Training And Education In Professional Psychology	1 (1.3)	2.3
5.	Women’s Health Issues	1 (1.3)	3.1
5.	World Psychiatry	1 (1.3)	79.7
	Total	29 (36.4)	43.8
	Average Impact Factor 2017–2019		4.4
	During the COVID-19 pandemic 2020–2022 (*n*=261)		
1.	Telemedicine And E Health	20 (7.7)	5.0
2.	Archives Of Clinical Neuropsychology	13 (5.0)	3.4
3.	Clinical Neuropsychologist	11 (4.2)	4.4
4.	Frontiers In Psychiatry	7 (2.7)	5.4
5.	Journal Of Telemedicine And Telecare	6 (2.3)	6.3
5.	Psychological Services	6 (2.3)	3.1
	Total	63 (24.2)	27.6
	Average Impact Factor 2020–2022		4.6

a Journals which did not have the Impact Factor stated on the journal’s website or Journal Citation Report (JCR), the CiteScore stated on the journal’s website was used.

### Analysis of countries

#### Co-authorship analysis active countries

eTable S2, Supplemental Digital Content 1, http://links.lww.com/MS9/A383 includes the top 5 active countries. The United States was ranked topmost where a maximum number of articles were published in both pre-COVID-19-related literature (*n*= 63; 78.75%) and during COVID-19-related literature (*n*=181; 69.34%), followed by Canada and Australia in pre-COVID-19 whereas UK and Australia in during COVID-19. In pre-COVID-19-related literature, strong collaboration among the countries were found in the United States (TLS=9), Australia (TLS=9), and Canada (TLS= 7). Whereas during COVID-19-related literature, strong collaboration among the countries were found in the United States (TLS= 21), Australia (TLS= 13), and United Kingdom (TLS= 8).

#### Distribution based on WHO region

**Figure 3:**
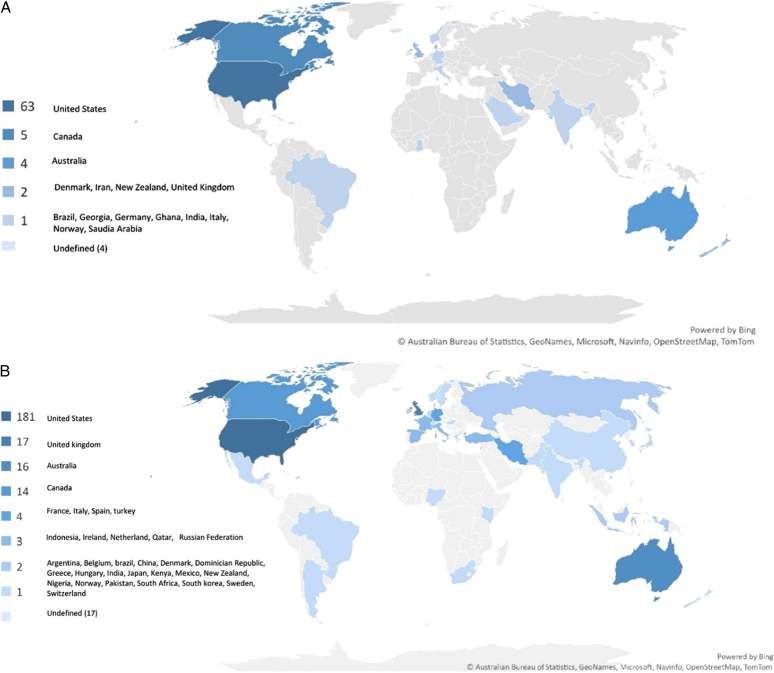
(A) Geographical distribution of the number of publications by countries based on WHO region before the COVID-19 pandemic (2017–2019); (B) Geographical distribution of the number of publications by countries based on WHO region during the COVID-19 pandemic (2020–2022).

Our results indicated that the Region of Americas (AMRO) had the highest contribution in both the pre-COVID-19 era (*n*=69; 86.25%) and during the COVID-19 era (*n*= 199; 76.24%), respectively. Whereas, the African region was the least contributing region in both pre-COVID-19 (*n*= 1; 1.25%) and during the COVID-19 era (*n*= 3; 1.14%) (Fig. 3A and B).

### Co-authorship analysis of authors

In eTable S3, Supplemental Digital Content 1, http://links.lww.com/MS9/A383, before the COVID-19 pandemic, a few authors contributed to one-fifth of all articles (*n*=16): Carpenter M. (*n*=4, 5%), Drouin M. (*n*=4, 5%), Shore J. H. (*n*=4, 5%), and Tosco T. (*n*=4, 5%). During the pandemic, author Lindsay J.A. published a higher number of articles (*n*=7, 2.68%). The mean h-index per total number of articles was higher before COVID-19 (4.64) compared to during COVID-19 (1.89). Before COVID-19, authors like Busch A.B, Carpenter M., Drouin M., Huskamp H.A., Mehrotra A., Toscos T., and Uscher-Pines L. had higher collaboration rates each (TLS=15) [eFigure S9 (A), Supplemental Digital Content 1, http://links.lww.com/MS9/A383]. During COVID- 19, Bunnell B.E., Huskamp H.A., Mehrotra A., and Paige S.R. had the greatest number of collaborations each (TLS=16) [eFigure S9 (B), Supplemental Digital Content 1, http://links.lww.com/MS9/A383].

### Analysis of publication and citations output

eFigure S7, Supplemental Digital Content 1, http://links.lww.com/MS9/A383 shows a gradual increase in studies from 2017 (*n*=21) to 2019 (*n*=33), almost doubling in 2020 (n=74), followed by a rapid decline in 2022 (n=69). The peak of publications was in 2021, and the lowest was in 2017.

eFig S8 indicates an increase in citations during the pandemic. It doubled from 2020 to 2021 (*n*=1480) and rose by approximately one-fifth from 2021 to 2022 (*n*=1840). The highest number of citations was in 2022, and the lowest was in 2017.

### Co-occurrence analysis of keywords

**Figure 4:**
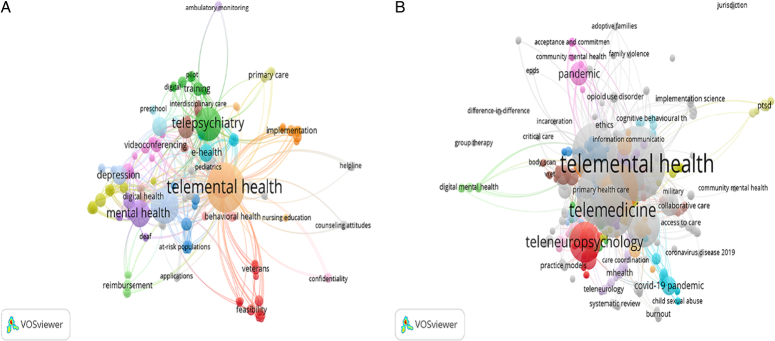
(A) Network visualization map for co-occurrence analysis of author keywords contributed to tele-neuropsychology and tele-mental health research publications before the COVID-19 pandemic (2017–2019); (B) Network visualization map for co-occurrence analysis of author keywords contributed to tele-neuropsychology and tele-mental health research publications during the COVID-19 pandemic (2020–2022).

Before the COVID-19 pandemic, the most common keywords were tele-MH (*n*=39, 48.8%), telemedicine (*n*=21, 26.3%), and telehealth (*n*=20, 25.0%). During the pandemic, the most common keywords were tele-MH (*n*=113, 43.3%), telehealth (*n*=84, 32.2%), and COVID-19 (*n*=67, 25.7%) (eTable S4, Supplemental Digital Content 1, http://links.lww.com/MS9/A383). The keyword clusters before and after the COVID-19 pandemic are shown in (Fig. 4A and B).

### Co-authorship analysis of affiliations

In eTable S5, Supplemental Digital Content 1, http://links.lww.com/MS9/A383, before the COVID-19 pandemic, Harvard Medical School had the highest number of publications (*n*=8, 10%). During the pandemic, Harvard Medical School remained a major contributor with 26 articles (9.96%). Before the pandemic, out of 222 organizations, 200 institutes with whom authors were affiliated (90.09%) had at least one collaboration (≥1 TLS). During the pandemic, out of 861 affiliated institutions, 829 institutions (96.28%) had at least one collaboration [eFigure S10 (A), Supplemental Digital Content 1, http://links.lww.com/MS9/A383]. The Rand Corporation had the highest number of collaborations before the pandemic (TLS=11), while Harvard Medical School had the highest number of collaborations during the pandemic (TLS=26) [eFigure S10 (B), Supplemental Digital Content 1, http://links.lww.com/MS9/A383].

### Analysis of the number of documents by top funding sponsors

eFigure S11 (A & B), Supplemental Digital Content 1, http://links.lww.com/MS9/A383 shows a higher proportion of publications supported by funding sponsors during the COVID-19 pandemic (*n*=181, 69.34%) compared to before the pandemic (*n*=42, 52.5%). The National Institute of Mental Health funded the highest number of documents before the pandemic (*n*=6, 7.50%), while the National Institutes of Health funded the most during the pandemic (*n*=13, 4.98%). During the pandemic, the National Institutes of Health, U.S. Department of Veterans Affairs (*n*=7, 2.68%), and U.S. Department of Health and Human Services (*n*=6, 2.30%) funded three times as many documents as before the pandemic (eTable S6, Supplemental Digital Content 1, http://links.lww.com/MS9/A383).

### Publication types, and subject areas

Before the COVID-19 pandemic, the most frequent publication types were reviews (*n*=27, 33.8%), surveys (*n*=14, 17.5%), and miscellaneous (*n*=13, 16.3%). During the COVID-19 pandemic, the most frequent publication types were reviews (*n*=61, 23.4%), miscellaneous (*n*=54, 20.7%), and surveys (*n*=44,16.9%) [eFigure S12 (A & B), Supplemental Digital Content 1, http://links.lww.com/MS9/A383]. In both pre-COVID-19 and during COVID-19 articles, medicine was the most common subject area (52% and 46%, respectively) [eFigure S13 (A & B), Supplemental Digital Content 1, http://links.lww.com/MS9/A383].

## Discussion

Our results demonstrate the greatest increase in publications and citations output from the year 2020 to 2021. Moreover, the average IF of journals publishing tele-NP and tele-MH research from 2020 to 2022 was higher than those from 2017 to 2019. This comparable increase in the average IF was likely due top- tier journals demonstrated a greater inclination to publish (tele-NP and tele-mental health tele-MH research, aligning with the increasing recognition of this treatment modality in the scientific community during the COVID-19 outbreak. Additionally, certain studies noted a positive impact on the IF of global health journals during the pandemic, potentially linked to the surge in citations for COVID-19-related research^12^. The IF, determined by the citation count within a specific timeframe, might have elevated due to the increased citation likelihood of COVID-19 studies, reflecting the heightened relevance and citation impact of pandemic-related research.

Our analysis highlights an increase in research on anxiety, depression, and trauma-related stress disorders during the pandemic, with heightened levels linked to financial strain, disruptions in education/employment, and loneliness^13^. The variation in these disorders may not be solely attributed to the pandemic timeline. It might be because certain disorders are conventionally managed with telehealth over others. Additionally, the increase in people’s intrusive traumatic re-experiencing during the pandemic is proposed to be related to the future rather than the past; indirectly via media coverage or exposure to infection rather than actual or threatened death, injury, or sexual violation (criterion A DSM-V)^14^. Thus, our results raise concerns regarding the relevance of DSM-V to the COVID-19 pandemic. The application of a more liberal interpretation of Criterion A will dilute the PTSD diagnosis, increase heterogeneity, confound case–control research, and create an overall sample pool with varying degrees of risk and vulnerability factors^15,16^. Acknowledging the unique challenges of tele-NP and tele-MH, clinicians should adopt a flexible approach to applying DSM-V criteria. Establishing guidelines for the ethical and effective use of DSM-V in virtual settings is essential for maintaining care standards.

Our analysis also shows that a greater number of journals are publishing tele-NP and tele-MH-related research, with greater frequency, during the COVID-19 pandemic than before. Telemedicine and E- health was found to be at the forefront because it was among the top five journals publishing articles in this regard both before and after the onset of the pandemic. The increase in research output following the COVID-19 outbreak reflects the growing interest and relevance of telemedicine for the delivery of mental health services. While medical care has been delivered remotely prior to the COVID-19 pandemic as well, the stringent lockdowns, mandatory social distancing, and increased patient load in hospitals after the coronavirus outbreak have caused many hospitals and clinics to expand their healthcare delivery by providing telehealth services^17^.

Our results show that during COVID-19, the number of published articles increased significantly, but the percentage of articles authored by top authors decreased. Before COVID-19, a smaller group of authors contributed to a greater proportion of articles (3.75–5%), but during COVID-19, top authors’ contributions ranged from 1.53 to 2.68%. The extensive social distancing protocols during the pandemic limited other opportunities to work in the field, leading medical professionals and students to engage in socially distant opportunities like research, resulting in a greater number of publications and decreased influence of top authors in the field.

Our bibliometric analysis assessed active countries based on WHO regions. During the COVID-19 pandemic, the United States, United Kingdom, Australia, and Canada had more tele-MH publications. Before the pandemic, the United States, Canada, and Australia were the top contributors. The Region of the Americas is the dominant region in publishing tele-MH literature, likely due to strong American collaboration, higher research funding allocation based on GDP, and European authors’ tendency to publish in American Journals^18^.

Publications by funding sponsors doubled during the pandemic compared to the pre-pandemic era. US-based funding agencies like The National Institutes of Health, the U.S. Department of Veterans Affairs, and the U.S. Department of Health and Human Services predominantly supported publications in both periods, highlighting potential funding disparities. Limited funding allocation in low-income and middle-income countries may be influenced by socioeconomic and political factors, including corruption, conflict, crime, and economic considerations^19^.

### Limitations

Despite our efforts, some limitations which are innate to bibliometric analysis remain. Firstly, we used a single database, SCOPUS. However, the fact that Scopus provides wider overall coverage as compared to Web of Science (WoS) was confirmed multiple times, both by early and the most recent content coverage comparisons. Generally, the content indexed in WoS and Scopus was also shown to be highly overlapping, with Scopus indexing a greater number of unique sources not covered by WoS^20^. The data search was completed in October 2022, which may not fully represent the entire COVID- 19 period (2019–2022). Some studies conducted before the pandemic were published later during the pandemic, impacting their accurate placement. However, this approach was intentional to reflect the increased acceptance of relevant articles during the COVID-19 era. Additionally, the analysis focused on specific study types for neuropsychology disorders and administration, potentially leading to data under or over-representation. Lastly, due to the exploratory nature of our analysis, we opted for a qualitative approach. Future research studies should consider employing quantitative analysis methods to enhance the robustness of these findings.

## Conclusion

Our bibliometric analysis delivers a comprehensive assessment of the altered trends pertaining to tele- NP and tele-MH since the onset of the COVID-19 pandemic. Trauma stressor-related disorders, anxiety, and depression were the most cited DSM-V disorders during the pandemic. There was an increase in global collaboration, particularly in the United States. The Americas contributed the most, while Africa had the least contribution. Before the pandemic, 90.09% of the 222 organizations had at least one collaboration among 200 affiliated institutes. During the pandemic, 96.28% of the 861 affiliated institutions engaged in at least one collaboration. Additionally, there was an increase in publications, citation count, and funding sponsors during the pandemic. Although our results strongly suggest the increased relevance of telemedicine, future studies need to incorporate patient expectation and satisfaction metrics for the telehealth modality.

## Ethics approval

Ethics committee approval is not required as there is no human or animal research.

## Consent

Not applicable.

## Source of funding

Not applicable.

## Author contribution

The contents of the paper were supervised and conceptualized by A.A. and R.Z. along with data analysis and drafting of the paper. The paper's drafting and analysis was assisted by K.A.K. and K.S. V.M. and S.I. critically revised the manuscript. H.F. assisted in data collection. All the authors approved the paper for final publication.

## Conflicts of interest disclosure

Not applicable.

## Research registration unique identifying number (UIN)

Not applicable.

## Guarantor

Dr Sufyan Ibrahim.

## Data availability statement

The original contributions presented in the study are included in the article. Supplementary material, further inquiries can be directed to the corresponding author.

## Provenance and peer review

Not commissioned, externally peer-reviewed.

## Supplementary Material
