# Supplementary material for: Application of tele-neuropsychology and tele-mental health before and during COVID-19 era: a bibliometric analysis
Source: Ann Med Surg (Lond). 2024 Feb 19;86(5):2777–85. doi: 10.1097/MS9.0000000000001822 (PMC11060194; doi:10.1097/MS9.0000000000001822)
Supplement: Supplementary file 1 [file ms9-86-2777-s001.docx]

**SUPPLEMETARY FILE FIGURE LEGENDS**

**eFigure S5: Type of Tele-Neuropsychology and Tele-Mental Health administered before (2017-2019) and during the COVID-19 pandemic (2020-2022).**

**eFigure S6: (A) Network visualization map for citation analysis of active journals on Tele-Neuropsychology and Tele-Mental health research publications before the COVID-19 pandemic (2017-2019); (B) Network visualization map for citation analysis of active journals on Tele-Neuropsychology and Tele-Mental health research publications after the COVID-19 pandemic (2020-2022).**

**eFigure S7: Number of publications on Tele-Neuropsychology and Tele-Mental Health before (2017-2019) and after (2020-2022) the COVID-19 pandemic.**

**eFigure S8: Number of citations on Tele-Neuropsychology and Tele-Mental Health before (2017-2019) and after (2020-2022) the COVID-19 pandemic.**

**eFigure S9: (A) Network visualization map for co-authorship analysis of authors that contributed to Tele-Neuropsychology and Tele-Mental health research publications before the COVID-19 pandemic (2017-2019); (B) Network visualization map for co-authorship analysis of authors that contributed to Tele-Neuropsychology and Tele-Mental health research publications during the COVID-19 pandemic (2020-2022).**

**eFigure S10: (A) Network visualization map for co-authorship analysis of affiliations that contributed to Tele-Neuropsychology and Tele-Mental health research publications before the COVID-19 pandemic (2017-2019); (B) Network visualization map for co-authorship analysis of affiliations that contributed to Tele-Neuropsychology and Tele-Mental health research publications during the COVID-19 pandemic (2020-2022).**

**eFigure S11: (A) Number of documents on Tele-Neuropsychology and Tele-Mental Health contributed by funding sponsors before the COVID-19 pandemic (2017-2019); (B) Number of documents on Tele-Neuropsychology and Tele-Mental Health contributed by funding sponsors after the COVID-19 pandemic (2020-2022).**

**eFig S12: (A) Type of Tele-Neuropsychology and Tele-Mental Health research published before the COVID-19 pandemic (2017-2019); (B) Publication Type of Tele-Neuropsychology and Tele-Mental Health research published during the COVID-19 pandemic (2020-2022).**

**eFigure S13: (A) Subject Areas that contributed to Tele-Neuropsychology and Tele-Mental Health before the COVID-19 pandemic (2017-2019); (B) Subject Areas that contributed to Tele-Neuropsychology and Tele-Mental Health during the COVID-19 pandemic (2020-2022).**

**eTable S2: (A) Top 5 active countries contributed on Tele-Neuropsychology and Tele-Mental health research publications before the COVID-19 pandemic (2017 – 2019) and during the COVID-19 pandemic (2020 - 2022).**

**eTable S3. Authors that contributed to Tele-Neuropsychology and Tele-Mental Health before (2017-2019) and during the COVID-19 pandemic (2020-2022).**

**eTable S4: Top 10 keywords that contributed on Tele-Neuropsychology and Tele-Mental Health research publications before (2017-2019) and during the COVID-19 pandemic (2019-2022).**

**eTable S5: Institutions that contributed to Tele-Neuropsychology and Tele-Mental Health before (2017-2019) and during the COVID-19 pandemic.**

**eTable S6: Top funding sources for Tele-Neuropsychology and Tele-Mental Health before (2017-2019) and during the COVID-19 pandemic (2020-2022).**

**eFigure S5: Type of Tele-Neuropsychology and Tele-Mental Health administered before (2017-2019) and during the COVID-19 pandemic (2020-2022).**

**
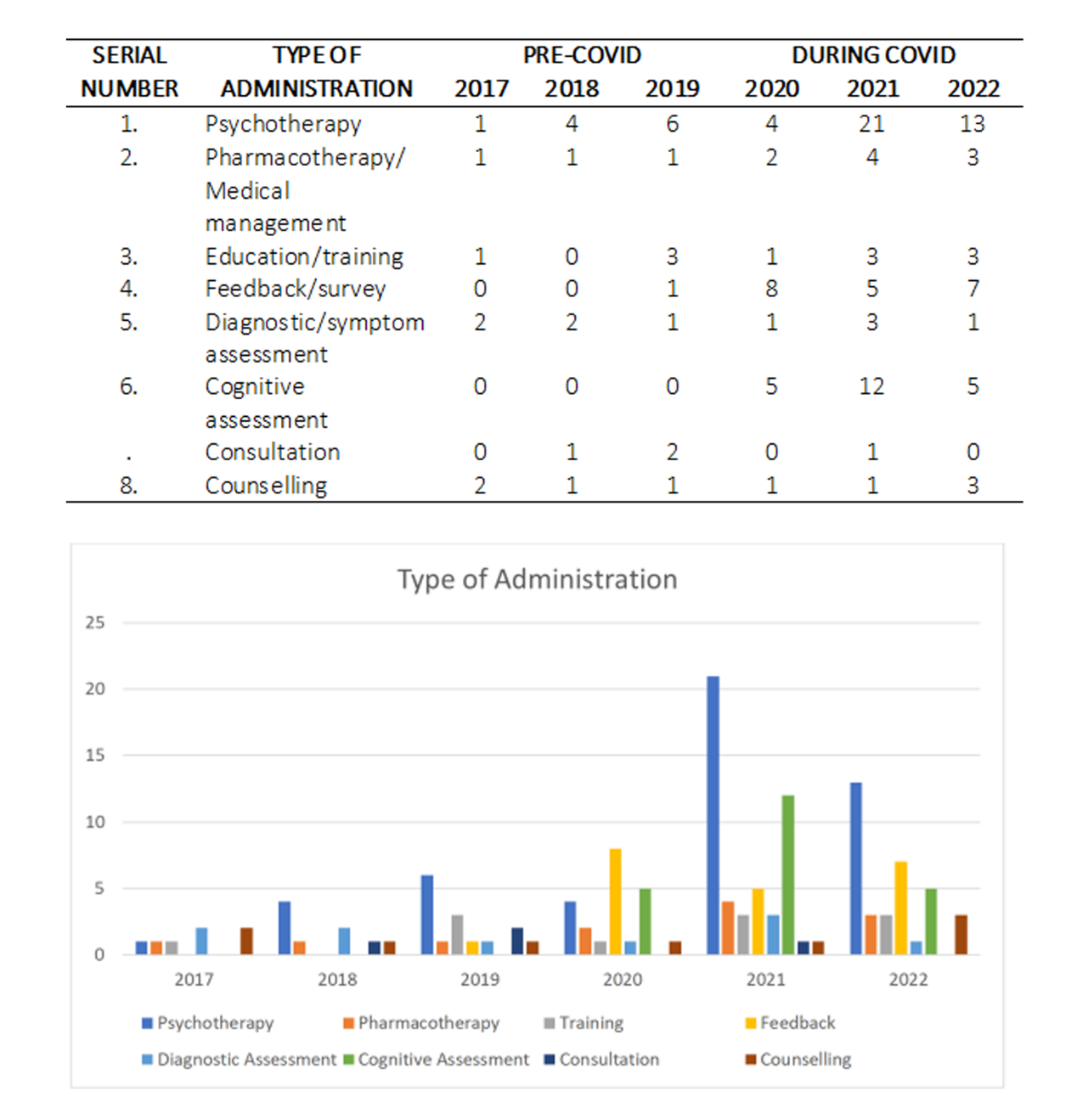
**

**
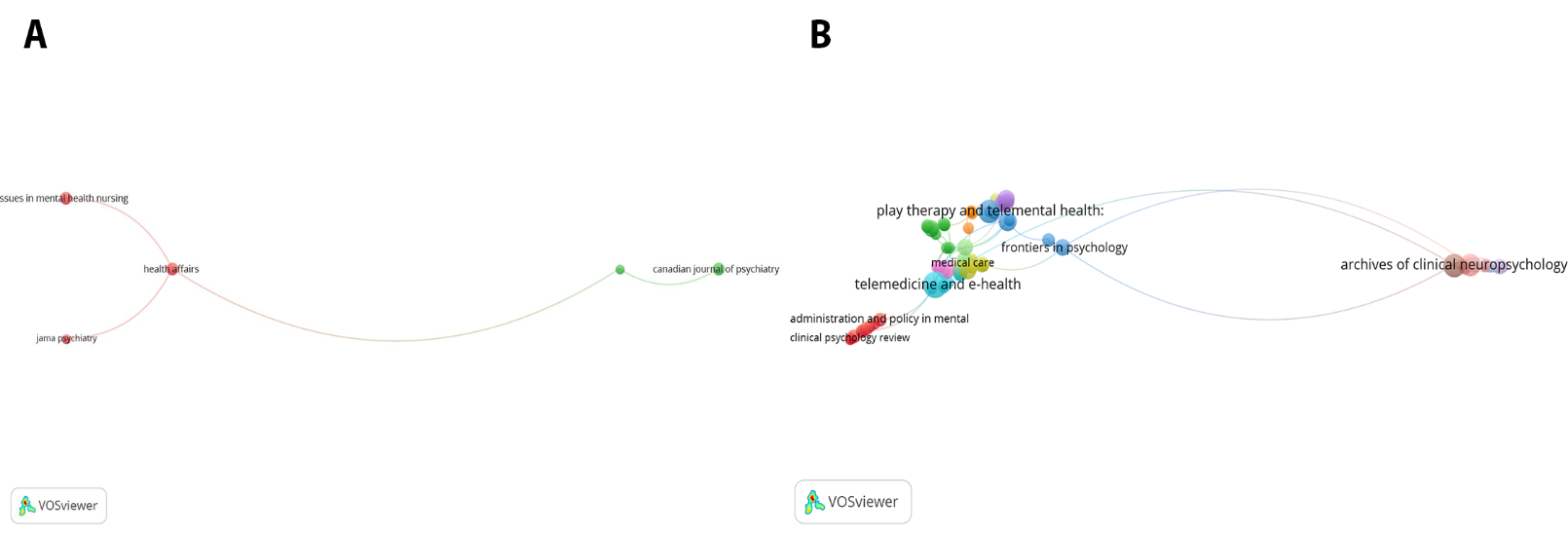
eFigure S6: (A) Network visualization map for citation analysis of active journals on Tele-Neuropsychology and Tele-Mental health research publications before the COVID-19 pandemic (2017-2019); (B) Network visualization map for citation analysis of active journals on Tele-Neuropsychology and Tele-Mental health research publications after the COVID-19 pandemic (2020-2022).**

**eFigure S7: Number of publications on Tele-Neuropsychology and Tele-Mental Health before (2017-2019) and after (2020-2022) the COVID-19 pandemic.**

**
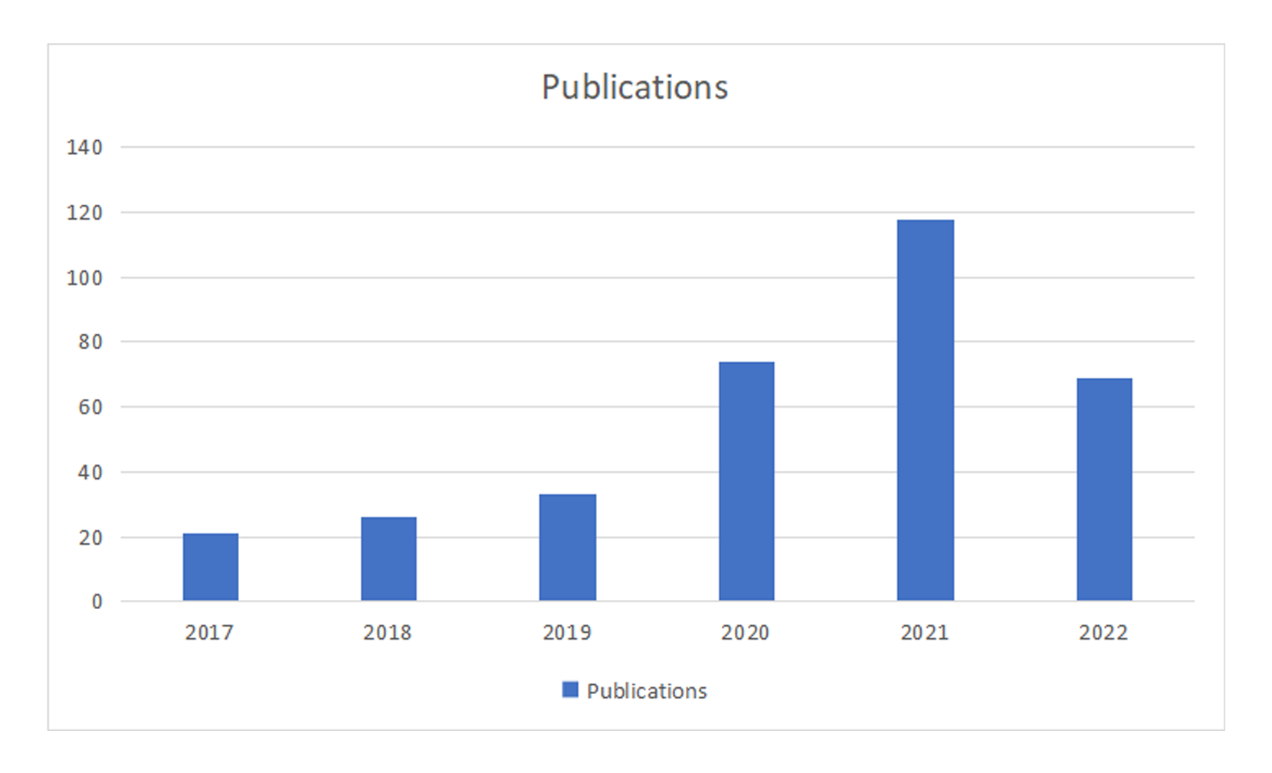
**

**eFigure S8: Number of citations on Tele-Neuropsychology and Tele-Mental Health before (2017-2019) and after (2020-2022) the COVID-19 pandemic.**

**
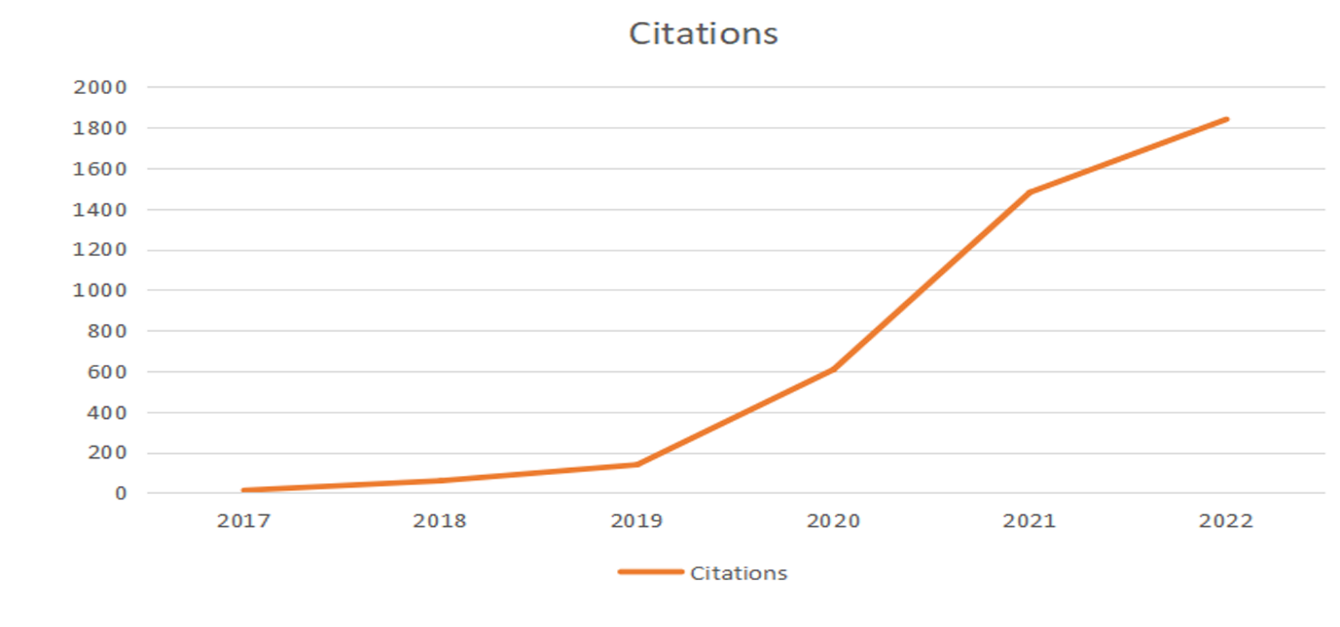
**

**
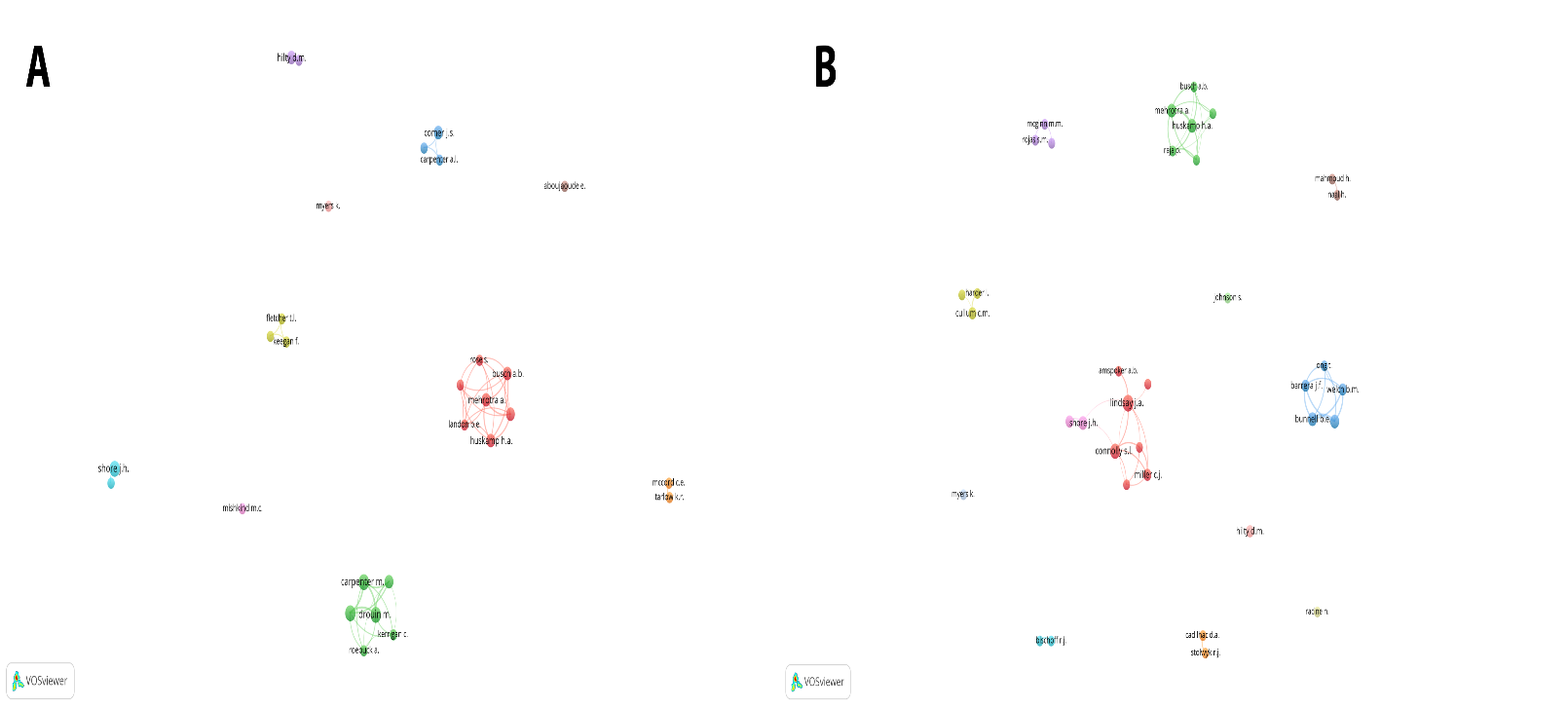
eFigure S9: (A) Network visualization map for co-authorship analysis of authors that contributed to Tele-Neuropsychology and Tele-Mental health research publications before the COVID-19 pandemic (2017-2019); (B) Network visualization map for co-authorship analysis of authors that contributed to Tele-Neuropsychology and Tele-Mental health research publications during the COVID-19 pandemic (2020-2022).**

**
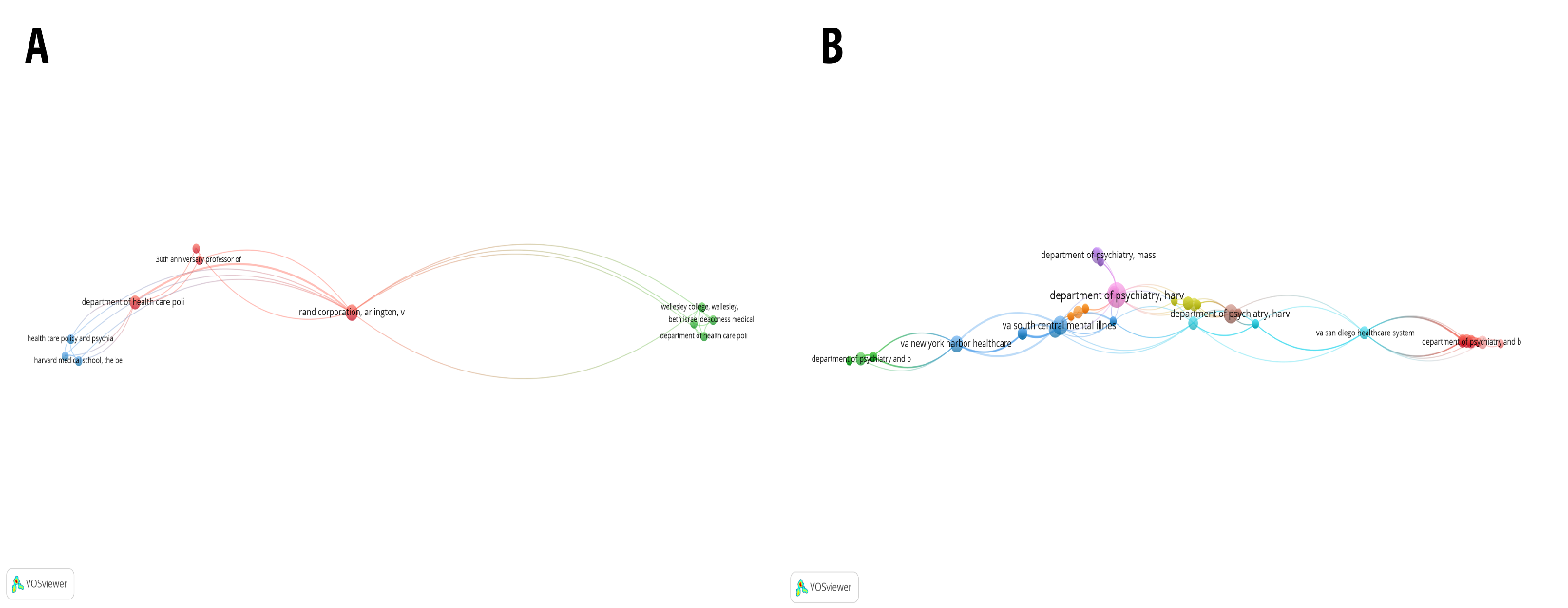
eFigure S10: (A) Network visualization map for co-authorship analysis of affiliations that contributed to Tele-Neuropsychology and Tele-Mental health research publications before the COVID-19 pandemic (2017-2019); (B) Network visualization map for co-authorship analysis of affiliations that contributed to Tele-Neuropsychology and Tele-Mental health research publications during the COVID-19 pandemic (2020-2022).**

**eFigure S11: (A) Number of documents on Tele-Neuropsychology and Tele-Mental Health contributed by funding sponsors before the COVID-19 pandemic (2017-2019); (B) Number of documents on Tele-Neuropsychology and Tele-Mental Health contributed by funding sponsors after the COVID-19 pandemic (2020-2022).**

**
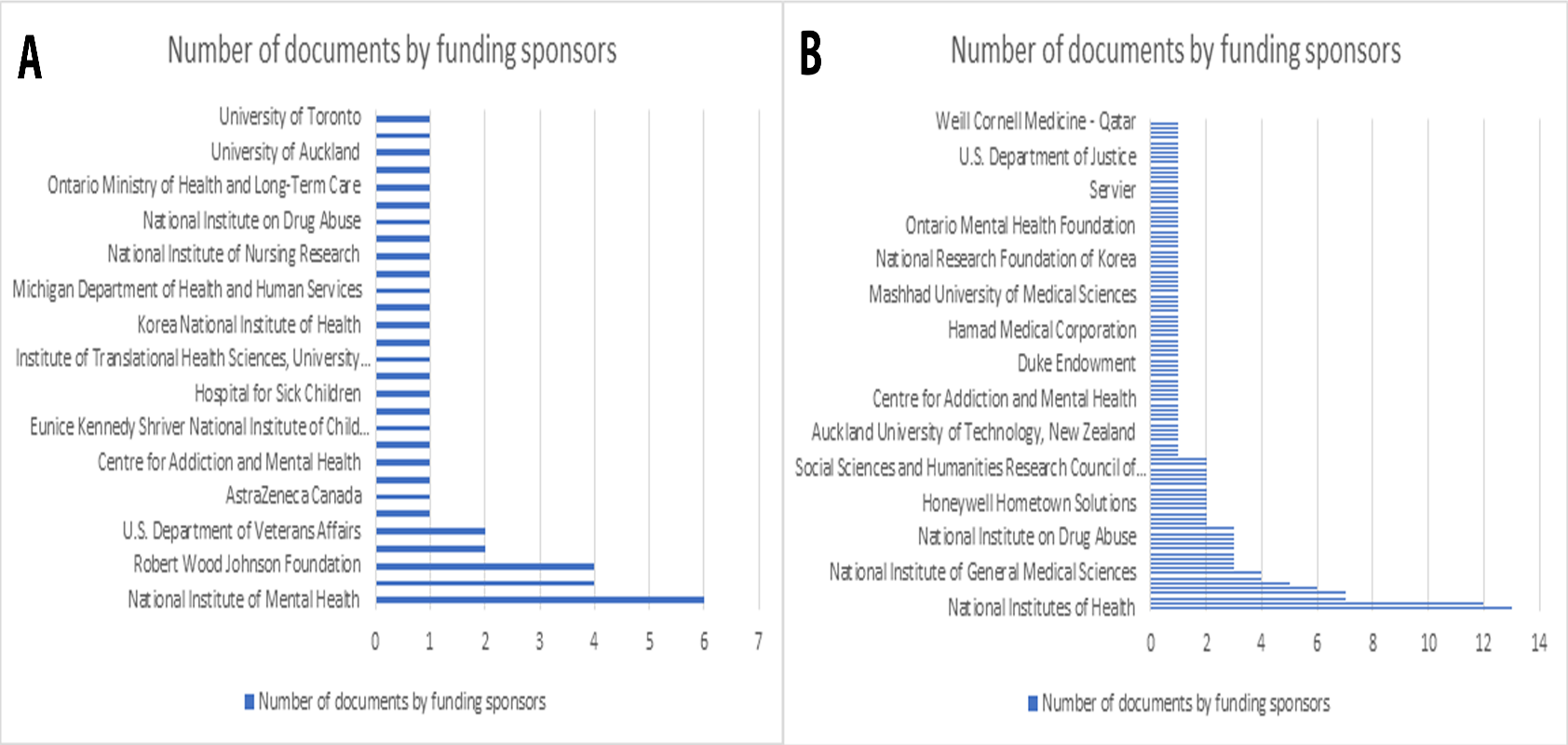
**

**
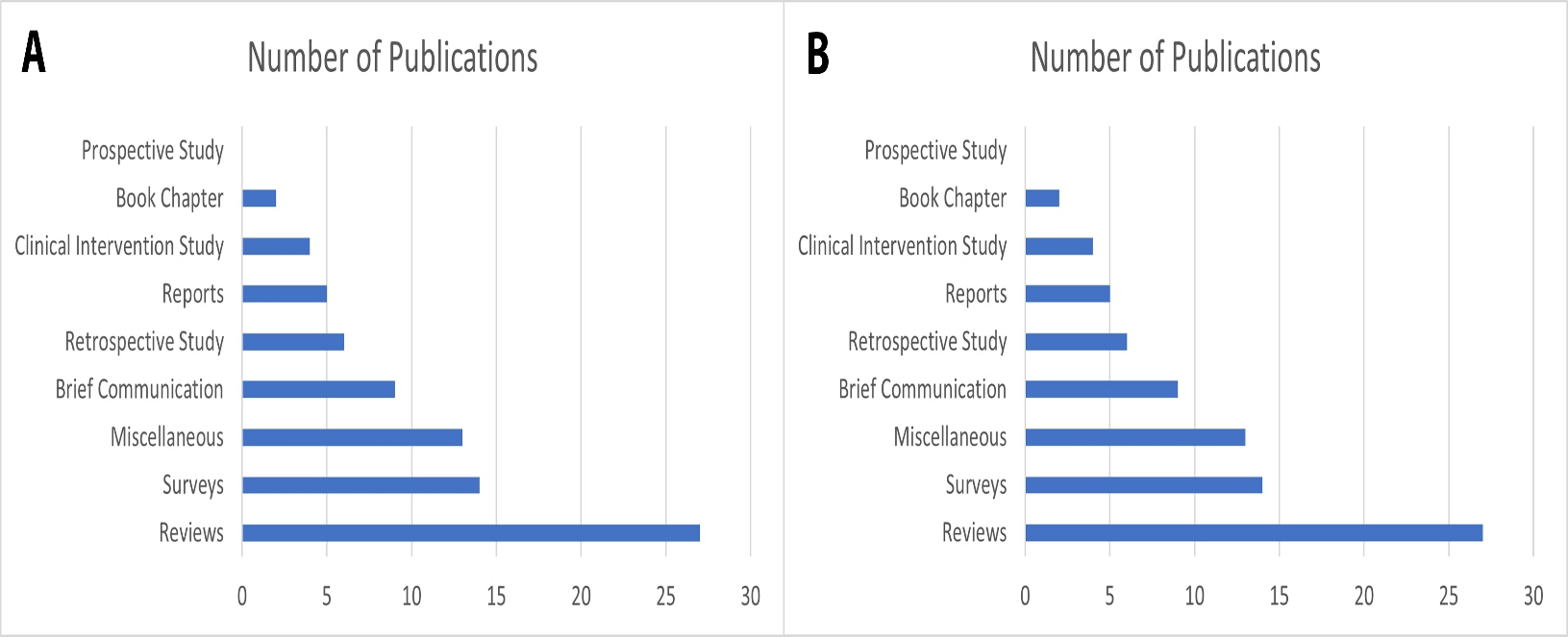
eFigure S12: (A) Type of Tele-Neuropsychology and Tele-Mental Health research published before the COVID-19 pandemic (2017-2019); (B) Publication Type of Tele-Neuropsychology and Tele-Mental Health research published during the COVID-19 pandemic (2020-2022).**

**Foot notes:**

*Reviews (Narrative reviews, scoping reviews, systematic reviews, and meta-analyses).*

*Reports (case reports and case studies).*

*Brief Communication (commentaries, editorials, correspondence, opinions, and Letter to the Editor).*

*Miscellaneous (Pilot studies, Feasibility studies, Conference papers, third space conversations, Quality improvement project, Quasi-experimental studies, Ecological, Autoethnographic, Framework analysis, Mixed methods and those that did not specify a study design).*

**
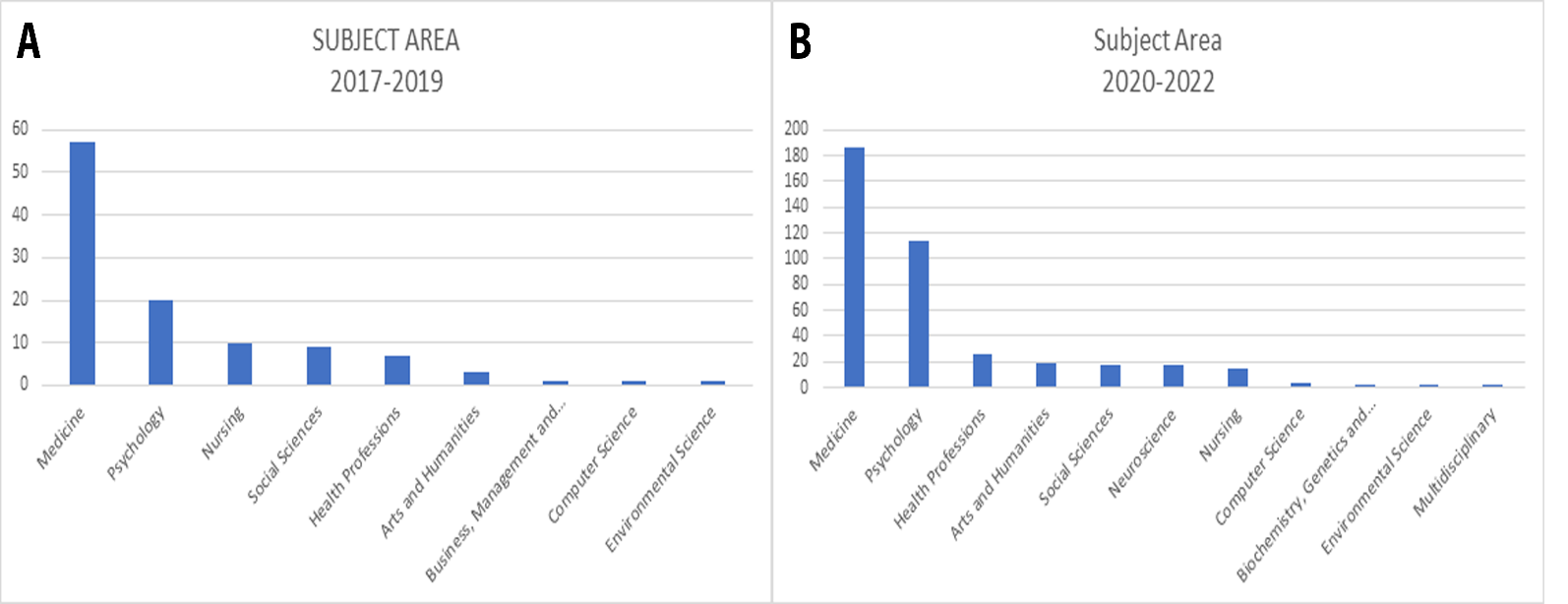
eFigure S13: (A) Subject Areas that contributed to Tele-Neuropsychology and Tele-Mental Health before the COVID-19 pandemic (2017-2019); (B) Subject Areas that contributed to Tele-Neuropsychology and Tele-Mental Health during the COVID-19 pandemic (2020-2022).**

**Table S2: Top 5 active countries contributed on Tele-Neuropsychology and Tele-Mental health research publications before (2017 – 2019) and during the COVID-19 pandemic (2020-2022).**

| RANK | ACTIVE COUNTRIES | NO. OF DOCUMENTS (%)  N=80 |
| --- | --- | --- |
| Before the COVID-19 Pandemic (2017-2019) | | |
| 1 | United States | 63 (78.75) |
| 2 | Canada | 05 (6.25) |
| 3 | Australia | 04 (5.00) |
| 4 | Denmark | 02 (2.50) |
| 4 | New Zealand | 02 (2.50) |
| 4 | United Kingdom | 02 (2.50) |
| 4 | Iran | 02 (2.50) |
| 5 | Georgia | 01 (1.25) |
| 5 | Brazil | 01 (1.25) |
| 5 | Ghana | 01 (1.25) |
| 5 | Germany | 01 (1.25) |
| 5 | India | 01 (1.25) |
| 5 | Italy | 01 (1.25) |
| 5 | Norway | 01 (1.25) |
| 5 | Saudi Arabia | 01 (1.25) |
|  |  |  |
|  | **During the COVID-19 Pandemic (2020-2022)**  **N=261** | |
| 1 | United States | 181 (69.34) |
| 2 | United Kingdom | 17 (6.51) |
| 3 | Australia | 16 (6.13) |
| 4 | Canada | 14 (5.36) |
| 5 | Germany | 04 (1.53) |
| 5 | Lebanon | 04 (1.53) |
| 5 | Iran | 04 (1.53) |

**Table S3: Authors that contributed to Tele-Neuropsychology and Tele-Mental Health before (2017-2019) and during the COVID-19 pandemic (2020-2022).**

| **Before the COVID-19 pandemic 2017-2019**  **(N=80)** | | | | **During the COVID-19 pandemic 2020-2022**  **(N=261)** | | | |
| --- | --- | --- | --- | --- | --- | --- | --- |
| Author | Number of Articles  N (%) | H-index | H-index per total articles | Author | Number of Articles  N (%) | H-index | H-index per total articles |
| Carpenter, M. | 4 (5) | 5 | 1.25 | Lindsay, J.A. | 7 (2.68) | 5 | 0.71 |
| Drouin, M. | 4 (5) | 16 | 4.00 | Connolly, S.L. | 6 (2.30) | 11 | 1.83 |
| Shore, J.H. | 4 (5) | 6 | 1.50 | Bunnell, B.E. | 5 (1.92) | 7 | 1.40 |
| Toscos, T. | 4 (5) | 13 | 3.25 | Huskamp, H.A. | 5 (1.92) | 13 | 2.60 |
| Busch, A.B. | 3 (3.75) | 9 | 3.00 | Mehrotra, A. | 5 (1.92) | 26 | 5.20 |
| Comer, J.S. | 3 (3.75) | 21 | 7.00 | Paige, S.R. | 5 (1.92) | 7 | 1.40 |
| Flanagan, M. | 3 (3.75) | 12 | 4.00 | Shore, J.H. | 5 (1.92) | 7 | 1.40 |
| Hilty, D.M. | 3 (3.75) | 17 | 5.67 | Barrera, J.F. | 4 (1.53) | 3 | 0.75 |
| Huskamp, H.A. | 3 (3.75) | 17 | 5.67 | Cullum, C.M. | 4 (1.53) | 1 | 0.25 |
| Mehrotra, A. | 3 (3.75) | 30 | 10.00 | Hilty, D.M. | 4 (1.53) | 11 | 2.75 |
| Uscher-Pines, L. | 3 (3.75) | 17 | 5.67 | Miller, C.J. | 4 (1.53) | 15 | 3.75 |
|  |  |  |  | Mishkind, M.C. | 4 (1.53) | 5 | 1.25 |
|  |  |  |  | Welch, B.M. | 4 (1.53) | 5 | 1.25 |

**Table S4: Top 10 keywords that contributed on Tele-Neuropsychology and Tele-Mental Health research publications before (2017-2019) and during the COVID-19 pandemic (2019-2022).**

| **Before the COVID-19 pandemic 2017-2019 (n=80)** | | |
| --- | --- | --- |
| **Keywords** | **Occurrence (%)** |  |
| Telemental health | 39 (48.8) |  |
| Telemedicine | 21 (26.3) |  |
| Telehealth | 20 (25.0) |  |
| Telepsychiatry | 19 (23.8) |  |
| Mental Health | 12 (15.0) |  |
| Telepsychology | 07 (8.8) |  |
| Depression | 06 (7.5) |  |
| Technology | 06 (7.5) |  |
| E-health | 05 (6.3) |  |
| Anxiety | 04 (5.0) |  |
| Youth | 04 (5.0) |  |
| E-Mental Health | 03 (3.8) |  |
| Mental Health Services | 03 (3.8) |  |
| Videoconferencing | 03 (3.8) |  |
| Training | 03 (3.8) |  |
|  |  |  |
| **During the COVID-19 pandemic 2020-2022 (n=261)** | | |
|  |  |  |
| Telemental health | 113 (43.3) |  |
| Telehealth | 84 (32.2) |  |
| Covid-19 | 67 (25.7) |  |
| Telemedicine | 59 (22.6) |  |
| Mental Health | 49 (18.8) |  |
| Telepsychiatry | 37 (14.2) |  |
| Teleneuropsychology | 34 (13.0) |  |
| Telepsychology | 14 (5.4) |  |
| Videoconferencing | 12 (4.6) |  |
| Veterans | 12 (4.6) |  |
| Pandemic | 11 (4.2) |  |

**Table S5: Institutions that contributed to Tele-Neuropsychology and Tele-Mental Health before (2017-2019) and during (2020-2022) the COVID-19 pandemic.**

| **Before the COVID-19 pandemic 2017-2019**  **(N=80)** | | **During the COVID-19 pandemic 2020-2022**  **(N=261)** | |
| --- | --- | --- | --- |
| **Affiliation** | **Number of Articles**  **N (%)** | **Affiliation** | **Number of Articles**  **N (%)** |
| Harvard Medical School | 8 (10.00) | Harvard Medical School | 26 (9.96) |
| Massachusetts General Hospital | 5 (6.25) | VA Medical Center | 22 (8.43) |
| Parkview Research Center | 4 (5.00) | Baylor College of Medicine | 11 (4.21) |
| VA Medical Center | 4 (5.00) | University of Washington | 11 (4.21) |
| Florida International University | 4 (5.00) | Michael E. DeBakey VA Medical Center | 9 (3.45) |
| University of Washington | 4 (5.00) | Medical University of South Carolina | 9 (3.45) |
| RAND Corporation | 3 (3.75) | Stanford University School of Medicine | 9 (3.45) |
| McLean Hospital | 3 (3.75) | VA Palo Alto Health Care System | 8 (3.07) |
| University of Toronto | 3 (3.75) | University of Colorado Anschutz Medical Campus | 8 (3.07) |
| Indiana University-Purdue University Indianapolis | 3 (3.75) | VA Boston Healthcare System | 8 (3.07) |
| University of Colorado Anschutz Medical Campus | 3 (3.75) | University of South Florida, Tampa | 7 (2.68) |
| Stanford University School of Medicine | 3 (3.75) | Massachusetts General Hospital | 7 (2.68) |
| Purdue University Fort Wayne | 3 (3.75) | Beth Israel Deaconess Medical Center | 7 (2.68) |

**Table S6: Top funding sources for Tele-Neuropsychology and Tele-Mental Health before (2017-2019) and during the COVID-19 pandemic (2020-2022).**

| Funding Sponsor | Frequency (%) |
| --- | --- |
|  |  |
| Before COVID-19 pandemic (2017-2019) | 80 |
| Funding sponsor | 42 (52.5) |
| No funding sponsor | 38 (47.5) |
|  |  |
| National Institute of Mental Health | 6 (7.5) |
| National Institutes of Health | 4 (5.0) |
| Robert Wood Johnson Foundation | 4 (5.0) |
| U.S. Department of Health and Human Services | 2 (2.5) |
| U.S. Department of Veterans Affairs | 2 (2.5) |
|  |  |
| During COVID-19 pandemic (2020-2022) | 261 |
| Funding sponsor | 181 (69.34) |
| No funding sponsor | 80 (30.65) |
|  |  |
| National Institutes of Health | 13 (4.98) |
| National Institute of Mental Health | 12 (4.6) |
| Health Resources and Services Administration | 7 (2.68) |
| U.S. Department of Veterans Affairs | 7 (2.68) |
| U.S. Department of Health and Human Services | 6 (2.3) |
| Health Services Research and Development | 5 (1.92) |
| Canadian Institutes of Health Research | 4 (1.5) |
| National Institute of General Medical Sciences | 4 (1.5) |
